# Supplementary material for: Transcranial Direct Current Stimulation Decreases P3 Amplitude and Inherent Delta Activity during a Waiting Impulsivity Paradigm: Crossover Study
Source: Brain Sci. 2024 Feb 7;14(2):168. doi: 10.3390/brainsci14020168 (PMC10887229; doi:10.3390/brainsci14020168)
Supplement: Supplementary file 1 [file brainsci-14-00168-s001.zip › brainsci-2836040-supplementary.pdf]

Supplementary Materials

Transcranial Direct Current Stimulation decreases P3 amplitude and inherent delta activity during a waiting impulsivity paradigm

S.M. 1. Study Design

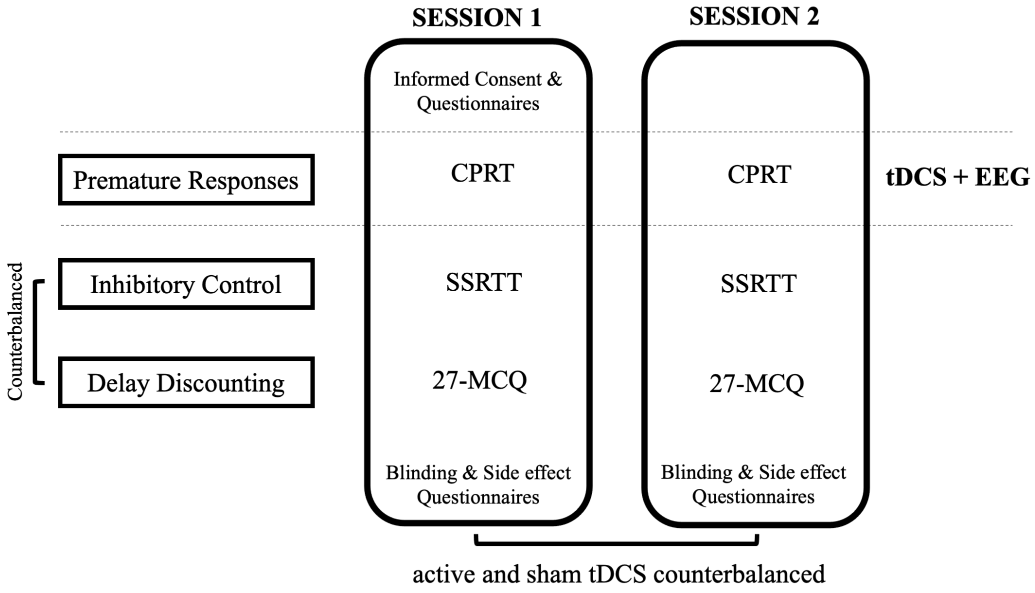

Figure S1. Overview of the experimental protocol in both sessions.

S.M. 2. Visual Analogue Scale

		Active tDCS			Sham tDCS		
		Pre	Post	Difference	Pre	Post	Difference
Visual Analogue Scale (VAS)	Fatigue	2.55 (1.89)	3.24 (1.89)	0.73 (1.52)	3.13 (2.29)	3.71 (2.29)	0.53 (1.52)
	Anxiety	1.45 (1.53)	0.86 (1.5)	-0.65 (1.25)	1.71 (1.95)	1.13 (1.68)	-0.58 (1.59)
	Sadness	0.68 (1.08)	0.43 (0.88)	-0.25 (0.78)	0.89 (1.31)	0.63 (1.01)	-0.25 (0.71)
	Agitation	1.55 (1.89)	1.41 (2.06)	-0.18 (1.74)	1.97 (2.26)	1.76 (2.09)	-0.2 (1.76)
	Sleepiness	2.13 (2.08)	2.68 (2.46)	0.68 (2.23)	2.13 (2.63)	2.97 (2.65)	0.75 (2.53)
	Itching	0.21 (0.99)	1.7 (2.34)	1.38 (1.98)	0.13 (1.06)	0.79 (1.33)	0.48 (1.6)
	Headache	0.47 (1.15)	0.73 (1.26)	0.23 (0.77)	0.47 (0.88)	0.74 (1.2)	0.25 (0.74)
	Another type of pain	0.29 (1.01)	0.32 (0.86)	0.03 (0.62)	0.24 (0.97)	0.24 (0.86)	0 (0.32)
	Tingling	0 (0)	0.92 (1.89)	0.85 (1.87)	0.03 (0.16)	0.47 (1.11)	0.43 (1.11)
	Metallic taste	0 (0)	0.03 (0.16)	0.03 (0.16)	0.08 (0.47)	0.16 (0.48)	0.08 (0.42)

Table S1. VAS of the side effects associated with tDCS in both sessions (Mean and SD)

S.M. 3. Blinding

Table S2. Blinding of tDCS in both sessions

Participant	Active Session		Sham Session	
	Guess	Confidence	Guess	Confidence
1	Active	1	NK	4
2	Active	3	Sham	3
3	Active	1	Active	1
4	Active	4	Sham	2
5	NK	-	Active	2
6	Active	3	NK	-
7	Active	1	Sham	1
8	Active	2	Active	2
9	Active	2	Missing Data	
10	Active	3	Sham	3
11	Active	3	NK	-
12	Sham	2	Sham	4
13	Sham	2	Active	2
14	Sham	3	Active	4
15	Active	4	Active	4
16	Active	3	Sham	4
17	Active	4	Active	4
18	Active	3	Active	3
19	Active	2	Active	2
20	Active	4	Sham	2
21	NK	-	Sham	1
22	NK	-	Active	2
23	Sham	2	Active	3
24	Sham	1	Active	2
25	Sham	2	Active	2
26	Sham	2	Sham	2
27	Active	2	Active	3
28	Sham	1	Sham	2
29	Sham	2	Active	3
30	Active	3	Sham	3
31	Sham	1	Sham	4
32	Active	1	Sham	2
33	Active	3	Active	3
34	Active	3	Active	3
35	NK	-	NK	-
36	NK	-	Sham	4

37	Active	4	Sham	3
38	Sham	1	Active	1
39	NK	-	NK	-
40	Active	2	Sham	2
Correct Guess	57.5		41.03	
Wrong Guess	27.5		46.15	
NK	15		12.82	

Legend: NK – "I do not know"; 0 – "No confident at all"; 1 – "Slightly confident"; 2 – "Moderately confident"; 3 – "Considerably confident"; 4 – "Extremely confident"

S.M. 4. Cued Premature Response Task

The reinforcement/punishment feedback was individualized to each participant according to the mean and variability of the release time (RT) observed in the last 10 trials of the baseline block (Figure 1.B), namely:

- Very fast responses: the RT was below -0.66 standard deviation (SD) of the baseline RT mean, which was reinforced with virtual 1€. In case of three successful consecutive trials, the feedback increased to 2€ as a reward for the "very fast responses".
- Fast responses: the RT was between -0.66 SD and +0.33 SD of the baseline RT mean and the participant received a virtual 0.5€.
- Slow responses: the RT was between +0.33 SD and +1SD of the baseline RT mean and the participant punishment was the loss of 0.5€.
- Very slow responses: the RT was above +1SD of the baseline RT mean and the participant would lose 1€.
- Premature responses: participant released the button before the target, which was neither reinforced, nor punished, and the feedback was instead "Continue" (i.e., please continue) in the native language.

The interval between the trial onset and the cue were very similar in both sessions, namely a mean of 1499.93 ms and a standard deviation of 9.49 ms in during the active tDCS session, and a mean of 1499.72 ms and a standard deviation of 10.25 ms in sham sessions. Likewise, the interval between the cue and the target had a mean of 1502.39 ms and a standard deviation of 47.16 ms in active sessions, and a mean of 1502.99 ms and a standard deviation of 49.75 ms in sham sessions.

Two participants were removed from the analysis because they did not correctly perform the baseline block and other outliers with values above/below three standard deviations from the mean were eliminated.

S.M. 5. EEG outliers

Seven participants were excluded from the EEG analysis due to: the saturation of the signal during the anodal tDCS session (4 participants), the number of EEG epochs in any condition was lower than 20 trials (2 participants), and one outlier with the difference of P3 amplitude between active and sham condition higher than 3 SD from the mean.

S.M. 6. Inter-Trial Phase Coherence (ITPC)

The inter-trial phase coherence (ITPC) was calculated given that the delta activity during cue-P3 was not reduced during active tDCS, as opposed to the target-P3. The ITC of delta band during the time-window of cue-P3 was marginally significant higher in sham in comparison with active tDCS ($t(32) = -1.74$, $p = 0.092$; Figure S1). Likewise, this statistically significant effect in delta ITC was also observed in target-

P3 (t(32) = -2.34, $p = 0.026$; Figure S2). This suggests that active tDCS increases the variability of the phase of delta activity within trials when compared to sham. However, the relation between tDCS and ITC is still not clear, one study has shown a synchronization of theta phase after frontal tDCS (Reinhart et al., 2015), whilst another study did not show any tDCS modulation in ITPC (Miyagishi et al., 2018). The literature is scarce about tDCS effects in ITC, nonetheless, the current findings are in line with the notion that the power and phase of EROs are distinct physiological processes (Burke et al., 2013; Buzsáki and Draguhn, 2004). Specifically, the decrease of cue-P3 amplitude during tDCS might be explained by higher variability in the

delta phase caused by tDCS, instead of the decrease in the delta evoked-power (together with phase variability) as occurred in target-P3.

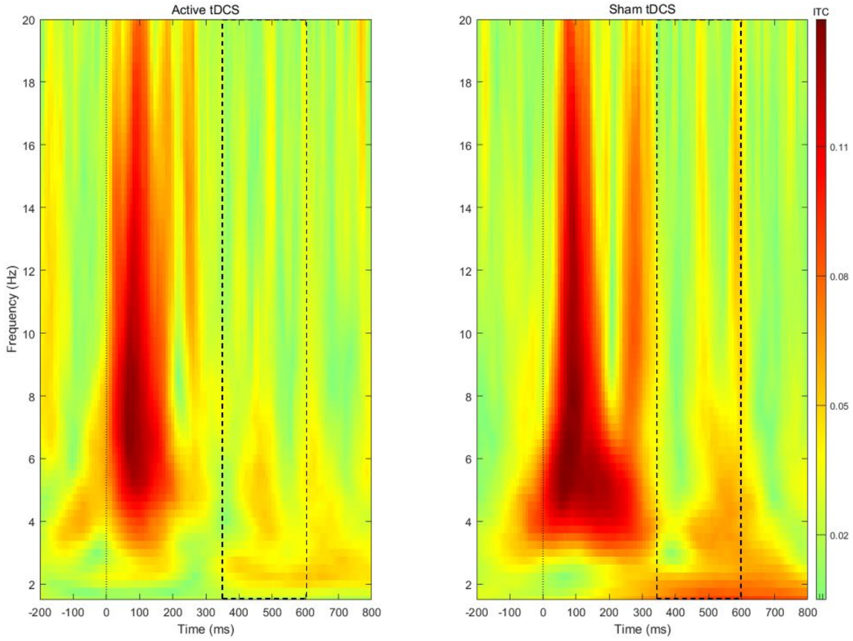

Figure S2. The ITPC results of cue-P3 at Pz electrode in the time-window of interest (dashed lines: 350 – 600 ms) between both tDCS condit

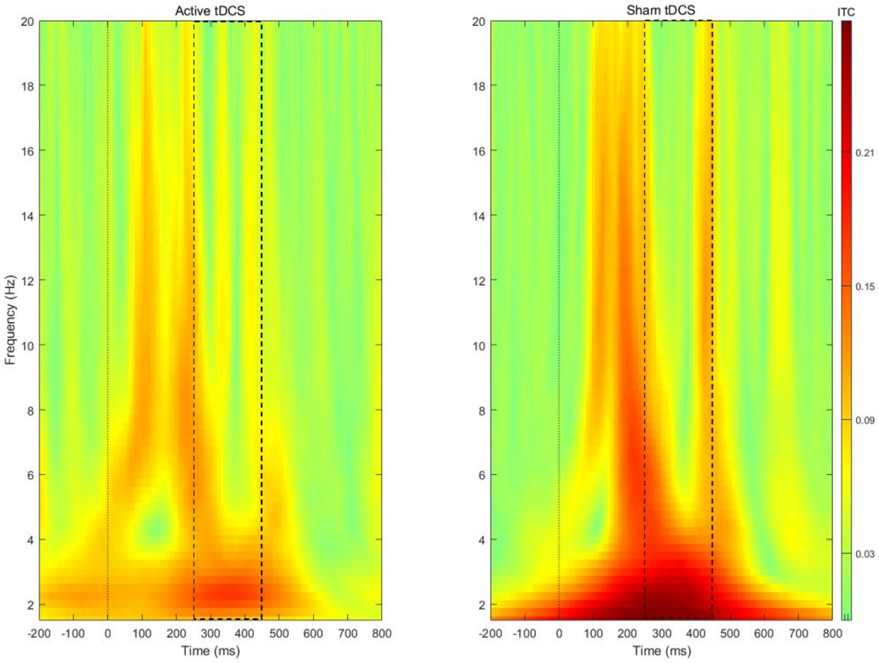

Figure S3. The ITPC results of target-P3 at Pz electrode in the time-window of interest (dashed lines: 250 – 450 ms) between both tDCS conditions.

Supplemental References

Burke, J.F., Zaghlou, K.A., Jacobs, J., Williams, R.B., Sperling, M.R., Sharan, A.D., Kahana, M.J., 2013. Synchronous and Asynchronous Theta and Gamma Activity during Episodic Memory Formation. J. Neurosci. 33, 292–304. https://doi.org/10.1523/JNEUROSCI.2057-12.2013

Buzsáki, G., Draguhn, A., 2004. Neuronal olscillations in cortical networks. Science (80-.). 304, 1926–1929. https://doi.org/10.1126/science.1099745

Miyagishi, Y., Ikeda, T., Takahashi, T., Kudo, K., Morise, H., Minabe, Y., Kikuchi, M., 2018. Gamma-band auditory steady-state response after frontal tDCS: A double-blind, randomized, crossover study. PLoS One 13, e0193422. https://doi.org/10.1371/JOURNAL.PONE.0193422

Reinhart, R.M.G., Zhu, J., Park, S., Woodman, G.F., 2015. Synchronizing theta oscillations with direct-current stimulation strengthens adaptive control in the human brain. Proc. Natl. Acad. Sci. U. S. A. 112, 9448–9453. https://doi.org/10.1073/PNAS.1504196112/-/DCSUPPLEMENTAL
